# Teenage-Onset Colorectal Cancers in a Digenic Cancer Predisposition Syndrome Provide Clues for the Interaction between Mismatch Repair and Polymerase δ Proofreading Deficiency in Tumorigenesis

**DOI:** 10.3390/biom12101350

**Published:** 2022-09-22

**Authors:** Esther Schamschula, Miriam Kinzel, Annekatrin Wernstedt, Klaus Oberhuber, Hendrik Gottschling, Simon Schnaiter, Nicolaus Friedrichs, Sabine Merkelbach-Bruse, Johannes Zschocke, Richard Gallon, Katharina Wimmer

**Affiliations:** 1 Institute of Human Genetics, Medizinische Universität Innsbruck, 6020 Innsbruck, Austria; 2 Medicover Humangenetik—Berlin-Lichtenberg, 10315 Berlin, Germany; 3 Institute of Pathology, University of Cologne, 50924 Cologne, Germany; 4 Translational and Clinical Research Institute, Faculty of Medical Sciences, Newcastle University, Newcastle upon Tyne NE2 4HH, UK

**Keywords:** digenic, colorectal cancer (CRC) in adolescents and young adults (AYA), POL-LYNCH, Lynch syndrome (LS), polymerase proofreading (PP), Pol δ, *POLD1*, *PMS2*, tumor mutational signature

## Abstract

**Simple Summary:**

Colorectal cancer (CRC) in adolescents and young adults (AYA) is rare. Genetic causes include autosomal recessive and dominant monogenic disorders due to pathogenic variants (PVs) in genes involved in DNA repair. However, the genetic etiology of the majority of AYA-CRC remains unidentified. In two teenage siblings with CRC, we show to our knowledge for the first time that AYA-CRC cases can be caused by digenic inheritance of each a heterozygous pathogenic variant (PV) in the mismatch-repair (MMR) gene *PMS2* and the proofreading polymerase (PP) Pol δ gene *POLD1*. With the aim to elucidate how the constitutional polymerase proofreading defect and the high propensity to MMR deficiency (MMRd) interact, we performed a comprehensive tumor analysis of the two siblings’ tumors. Results indicate that tumorigenesis is initiated by MMRd and the inherited *POLD1* PV contributes to fast tumor progression reflected by an ultra-high tumor mutational burden (TMB) and specific mutational signatures.

**Abstract:**

Colorectal cancer (CRC) in adolescents and young adults (AYA) is very rare. Known predisposition syndromes include Lynch syndrome (LS) due to highly penetrant *MLH1* and *MSH2* alleles, familial adenomatous polyposis (FAP), constitutional mismatch-repair deficiency (CMMRD), and polymerase proofreading-associated polyposis (PPAP). Yet, 60% of AYA-CRC cases remain unexplained. In two teenage siblings with multiple adenomas and CRC, we identified a maternally inherited heterozygous *PMS2* exon 12 deletion, NM_000535.7:c.2007-786_2174+493del1447, and a paternally inherited *POLD1* variant, NP_002682.2:p.Asp316Asn. Comprehensive molecular tumor analysis revealed ultra-mutation (>100 Mut/Mb) and a large contribution of COSMIC signature SBS20 in both siblings’ CRCs, confirming their predisposition to AYA-CRC results from a high propensity for somatic MMR deficiency (MMRd) compounded by a constitutional Pol δ proofreading defect. COSMIC signature SBS20 as well as SBS26 in the index patient’s CRC were associated with an early mutation burst, suggesting MMRd was an early event in tumorigenesis. The somatic second hits in *PMS2* were through loss of heterozygosity (LOH) in both tumors, suggesting PPd-independent acquisition of MMRd. Taken together, these patients represent the first cases of cancer predisposition due to heterozygous variants in *PMS2* and *POLD1.* Analysis of their CRCs supports that *POLD1*-mutated tumors acquire hypermutation only with concurrent MMRd.

## 1. Introduction

Colorectal cancer (CRC; MIM 114500) is the third most common cancer in countries of the Western world [1], with a median age at diagnosis of approximately 70 and 60 years for colon and rectal cancer, respectively [2]. CRC is rare in adolescents and young adults (AYA), with approximately 0.03% and 0.1% of CRCs being diagnosed in patients under the age of 20 years and 25 years, respectively, and is often more aggressive than in elderly patients [3,4]. An inherited CRC predisposition syndrome is found in about 35% and 40% of CRC patients diagnosed under the age of 35 and 25 years, respectively, which is more than 10 times more frequent than in the overall CRC patient population [4,5,6,7]. Lynch syndrome (LS; MIM 120435) is the most common cancer predisposition syndrome (CPS) found in AYA-CRC, with 23–29% of cases being attributable to LS. Predominantly (70–100%), LS AYA-CRC is caused by highly penetrant heterozygous germline pathogenic variants (PVs) in *MLH1* (MIM *120436) or *MSH2* (MIM *609309) [4,7,8]. In addition, a study by Fernandez-Rozadilla et al. indicates that low-risk genetic modifiers of CRC may contribute to very severe LS phenotypes with CRC diagnoses in childhood [9]. The same might be true for familial adenomatous polyposis (FAP; MIM 175100) [10], representing the second most common CPS in AYA-CRC [4,7]. A study investigating AYA-CRC patients without indication for LS and FAP revealed heterozygous germline (likely) PVs ((L)PVs) in additional cancer-predisposing genes, including *TP53* (MIM *191170), *BRCA2* (MIM *600185), *PALB2* (MIM *610355), *NF1* (MIM *613113), *MUTYH* (MIM *604933) and *MSH3* (MIM *600887) and a homozygous variant in *BLM* (MIM *604610) in 21% of patients [11], but most of these germline (L)PVs are unlikely to represent a monogenic predisposition to AYA CRC [12].

Constitutional mismatch-repair deficiency (CMMRD; MIM 276300), caused by bi-allelic germline PVs in a mismatch-repair (MMR) gene, was found in 1.0–2.7% of AYA-CRC cases [4,7]. The tumor spectrum of this very rare but highly penetrant pediatric and AYA CPS includes LS-associated carcinomas as well as hematological malignancies and brain tumors [13]. According to our current knowledge, it may be assumed that all CMMRD patients who reach AYA age will eventually develop colonic adenomas, frequently with high-grade dysplasia, and/or carcinomas, which often occur meta- and/or synchronously. CRC is diagnosed with a median age of onset of approximately 16 years in CMMRD patients [13]. In addition, nearly all CMMRD patients have characteristic, non-malignant clinical features. Of these, multiple café-au-lait macules (CALMs) and other alterations of skin pigmentation are the most prevalent [14]. Of note, the majority of both CMMRD syndrome in general and CMMRD-associated AYA-CRC are caused by bi-allelic germline PVs in *PMS2* (MIM *600259) [4,7,13].

Polymerase proofreading-associated polyposis (PPAP; MIM 615083 and 612591) is caused by *POLE* (MIM *174762) and *POLD1* (MIM *174761) exonuclease domain PVs and is characterized by the development of polyposis and CRC, as well as other cancers, in adulthood with a median age at CRC diagnosis of 41 and 43 years for *POLD1* PV and *POLE* PV carriers, respectively [15]. Several AYA-CRC patients, aged between 21 and 34 years, have been identified in families with classical PPAP [15]. Furthermore, we previously identified de novo germline *POLE* PVs in three CRC patients aged 13, 14, and 20 years who had a CMMRD-like phenotype [16]. As these three *POLE* PVs were so far found only as somatic mutations in ultra-mutated (>50 Mut/Mb) tumors but not as germline variants in PPAP patients, we speculated that they have a stronger “mutator” effect than known PPAP-causing PVs and, therefore, cause a more severe phenotype with CRC and/or brain tumors in childhood and adolescence as well as CMMRD-like non-malignant features [16,17]. Interestingly, somatic *POLE* PVs are also enriched in AYA-CRC (11% in AYA-CRCs vs. 3% in CRCs of patients aged ≥60 years) [4,18].

Approximately 60% of AYA-CRC cases remain unexplained. Here, we describe two teenage siblings with polyposis and CRC in whom we initially suspected CMMRD but found a novel digenic CPS caused by heterozygous germline PVs in *PMS2* and *POLD1*. To elucidate how the resulting constitutional polymerase proofreading defect and the high propensity to MMR deficiency (MMRd) interact in tumorigenesis, we performed a comprehensive molecular analysis of the patients’ tumors. The results indicate that at least the index patient’s CRC developed along the most common LS-tumorigenesis pathway with polymerase proofreading (PP) deficiency (PPd)-independent acquisition of MMRd being an early event and the constitutional PP defect promoting tumor progression in MMRd cells. These findings support that tumors with *POLD1* PVs acquire hypermutation only with concurrent MMRd [19].

## 2. Materials & Methods

### 2.1. Ethics

Informed consent was obtained from all family members to participate in this study and present their data.

### 2.2. Immunohistochemistry

Immunohistochemical staining (IHC) was performed using a Ventana Bench Mark Ultra automated staining system (Roche, Mannheim, Germany) according to the manufacturers’ protocols. The following antibodies were used: MLH1 (Clone M1, Ventana, Roche, Mannheim, Germany, mouse anti-human, monoclonal, dilution: ready to use), MSH2 (Clone G219-1129, Ventana, Roche, Mannheim, Germany, mouse anti-human, monoclonal, dilution: ready to use), MSH6 (KlonSP93, Ventana, Roche, Mannheim, Germany, rabbit anti-human, monoclonal, dilution: ready to use), and PMS2 (Clone A16-4, Ventana, Roche, Mannheim, Germany, rabbit anti-human, monoclonal, dilution: ready to use). For antigen retrieval, sections were incubated with EDTA for 32 min (MSH2), 64 min (MLH1, MSH6) and 72 min (PMS2). Incubation time with the primary antibody was 32 min. Slides were counterstained with Mayer’s hematoxylin and mounted. IHC results were visualized using the OptiView DAB IHC detection kit (Roche, Mannheim, Germany) according to the manufacturers’ protocols.

### 2.3. Tumor Microsatellite Instability (MSI) Analysis

Tumor areas were marked by an experienced pathologist on a hematoxylin and eosin-stained slide. Corresponding unstained tumor and paired normal tissues were macro dissected from formalin-fixed, paraffin-embedded (FFPE) 10 µm thick tissue sections. After overnight digestion with Proteinase K, DNA extraction was performed with the Maxwell 16 FFPE Plus Tissue LEV DNA Purification Kit (Promega, Mannheim, Germany) on the Maxwell 16 (Promega, Mannheim, Germany) following the manufacturer’s instructions as described before [20]. An in-house PCR protocol, including primers for the mononucleotide markers BAT25 and BAT26 as well as the dinucleotide markers D5S346, D2S123 and D17S250, was performed with paired tumor and normal tissue DNA samples using the Platinum Taq Polymerase (Invitrogen (Fisher Scientific), Berlin, Germany) (Table S3). For evaluation, PCR was followed by fragment length analysis on an ABI PRISM 3500 Genetic Analyzer and analyzed with the GeneMapper 4.1 analysis tool (Applied Biosystems (Fisher Scientific, Berlin, Germany)).

### 2.4. DNA Extraction for Sequencing

Tumor DNA was extracted from FFPE tissues using the QIAamp DNA FFPE Tissue Kit (Qiagen, Hilden, Germany) according to the manufacturer’s recommendation. Blood DNA was extracted from whole blood using the Gentra Puregene Blood Kit (Qiagen, Hilden, Germany) or the QIAsymphony DSP DNA Kits (Qiagen, Hilden, Germany) according to the manufacturers’ protocols.

### 2.5. RNA Extraction

RNA was extracted from short-term cultured lymphocytes treated with puromycin as described in Etzler et al. [21], using the RNeasy Kit (Qiagen, Hilden, Germany).

### 2.6. Constitutional MSI Analysis

Constitutional MSI analysis was performed on blood DNA using the amplicon sequencing-based MSI assay developed by Gallon et al. [22]. Twenty-four mononucleotide repeat MSI markers are amplified in multiplex using single molecule molecular inversion probes [23]. Amplicons were purified using Ampure XP Beads (Beckman Coulter, High Wycombe, UK) following the manufacturer’s protocols, diluted to 4 nM using 10 mM Tris-HCl (pH8.5), and pooled to create a sequencing library. The library was sequenced to a target depth of 5000 reads/marker/sample using a MiSeq (Illumina, Cambridge, UK) following the manufacturer’s protocols and using custom sequencing primers [23]. Each sample had an MSI score generated using a custom bioinformatics pipeline [22]. Reads were aligned to human reference genome GRCh37 hg19 using BWA mem [24], and R (https://cran.r-project.org/ (accessed on 22 June 2020)) was used to extract the frequency of different length alleles in each MSI marker with reduced PCR and sequencing error using molecular barcodes. For each sample, MSI marker reference allele frequencies (RAFs) were compared to RAFs of a reference set of control (non-CPS) blood samples to generate an MSI score using R (https://cran.r-project.org/ (accessed on 22 June 2020)). MSI scores > 2 indicate increased MSI and, therefore, MMRd.

### 2.7. Multiplex-Ligation-Dependent Probe Amplification Analysis

Multiplex-ligation-dependent probe amplification (MLPA) was performed with the SALSA MLPA-Kit P008-C1-02 (MRC Holland, Amsterdam, The Netherlands) according to the manufacturer’s recommendation using 6 control DNAs, known to each carry two copies of *PMS2*- and *PMS2CL*-derived sequences as described in Wernstedt et al. [25]. Electrophoretic separation and quantification of amplified products were performed with the ABI PRISM 3730xl Genetic Analyzer (Applied Biosystems (Fisher Scientific), Vienna, Austria). Data were analyzed with the SeqNext version 26 software (JSI medical systems).

### 2.8. Transcript Analysis

RNA was transcribed to cDNA using the SuperScript First-Strand Synthesis System for RT-PCR (Thermo Fisher (Fisher Scientific), Vienna, Austria) according to the manufacturer’s protocol. The complete coding sequence of the *PMS2* transcript was sequenced in 8 overlapping sequencing reactions using the ABI PRISM 3730xl Genetic Analyzer (Applied Biosystems (Fisher Scientific), Vienna, Austria) as described elsewhere [21]. Data were analyzed with the SeqNext version 26 software (JSI medical systems, Ettenheim, Germany).

### 2.9. Deletion-Spanning PCR and Sequencing

*PMS2* exon 12 deletion spanning PCR was performed with an unspecific forward primer located in intron 11, a gene-specific reverse primer in intron 12 and the Phusion HF-DNA polymerase (Biozym, Vienna, Austria). Together, these primers (Table S3) generate a deletion-specific PCR product of 1256 bp length in contrast to the 2703 bp long wild-type product. The amplicon was Sanger-sequenced with the ABI PRISM 3730xl Genetic Analyzer (Applied Biosystems (Fisher Scientific), Vienna, Austria). Data were analyzed with the SeqNext version 26 software (JSI medical systems, Ettenheim, Germany) and the Sequence Scanner Software v1.0 (Applied Biosystems (Fisher Scientific), Vienna, Austria).

### 2.10. Determination of Constitutional Variants by Panel Next-Generation Sequencing (NGS) of Blood DNA

To detect constitutional variants, massive-parallel sequencing was performed using the TruSight Cancer Sequencing Panel (Illumina, Eindhoven, The Netherlands) according to the manufacturer’s recommendations and sequenced on a MiSeq using 2 × 150 bp ‘paired-end reads’ and the MiSeq Flowcell and Reagent Kits v2 (Illumina, Eindhoven, The Netherlands). Sequences were aligned to the human reference genome GRCh37 hg19 and variants were called using the SeqNext version 26 software (JSI medical systems, Ettenheim, Germany). Variants with a variant allele frequency (VAF) of at least 10% affecting nucleotides with a read depth of at least 20 were classified according to the consensus recommendations of the American College of Medical Genetics [26] and the Mismatch Repair Gene Variant Classification Criteria v2.4 [27].

### 2.11. Determination of Somatic Variants by Whole-Exome NGS of Tumor and Blood DNA

For detection of somatic variants, libraries of tumor and blood DNA were prepared according to the Twist Library Preparation Protocols “Enzymatic Fragment and Twist Universal Adapter System” and “Twist Target Enrichment Protocol” and hybridized with a Twist Comprehensive Exome + Mitochondrial Panel (Twist Bioscience, San Francisco, CA, USA). Fragmentation time was set to 4 min for blood-derived and 2 min for tumor-derived samples. Massive-parallel sequencing was performed on a NextSeq2000 using 2 × 150 bp paired-end reads and P3 Flowcell and Reagent Kits (Illumina, Eindhoven, The Netherlands). DNA from tumor tissue and blood were sequenced separately. Sequences were aligned to the human reference genome b37/hg19 (GATK Resource Bundle). VCF files of somatic variants were generated using the GATK Best Practices-based Somatic Short Variant Discovery Pipeline of Tomas Bencomo (https://github.com/tjbencomo/ngs-pipeline (accessed on 29 July 2021), https://gatk.broadinstitute.org/hc/en-us/articles/360035894731-Somatic-short-variant-discovery-SNVs-Indels (accessed on 29 July 2021)) in paired mode [28,29,30,31,32,33,34,35]. Additional variant filtering and selection were performed using the VarSeq 2.2.4 software (Golden Helix). Variants with a VAF below 5% and/or at a position with a read depth below 20 were excluded. For the separate analysis of early and late mutational events of the patient’s tumor, somatic variants located on chromosomes 15, X or Y were omitted to prevent distortion of VAF-specific signatures since these chromosomes are not in the diploid state in neoplastic cells (Figure S2A). Equally, for the separate analysis of early and late mutational events of the sister’s tumor, somatic variants located on chromosomes 5, 7, 8, 13, 18, 19, and X were omitted (Figure S2B). Variants with the Flag “Variant is a short tandem repeat” determined by the VarSeq 2.2.4 software (Golden Helix) were considered short tandem repeat variants. Variants are classified according to the consensus recommendations of the American College of Medical Genetics [26] and the Mismatch Repair Gene Variant Classification Criteria v2.4 [27].

### 2.12. Calculation of Tumor Mutational Burden

Somatic variants that passed the quality filter applied as described in the section “Determination of somatic variants by whole-exome NGS of tumor and blood DNA” were used to calculate the tumor mutational burden (TMB) as Mut/Mb. According to the manufacturer, the Twist Comprehensive Exome + Mitochondrial Panel (Twist Bioscience, San Francisco, CA, USA) covers 36.8 Mb.

### 2.13. Mutational Signature Analysis

Tumor mutational signature analysis of all somatic variants passing the quality filter was performed using the SigProfiler bioinformatics tools v3.2 (SigProfilerMatrixGenerator and SigProfilerExtractor) with default settings and the COSMIC mutational signatures data files v3.2 for GRCh37 (https://cancer.sanger.ac.uk/signatures/tools/ (accessed on 4 March 2022)). For the separate analysis of variants representing early and late events, non-diploid chromosomes were excluded, as described in the section “Determination of somatic variants by whole-exome NGS of tumor and blood DNA.” To avoid bias, we introduced ploidy as described.

## 3. Results

### 3.1. Clinical History of Two AYA-CRC Siblings

**Figure 1:**
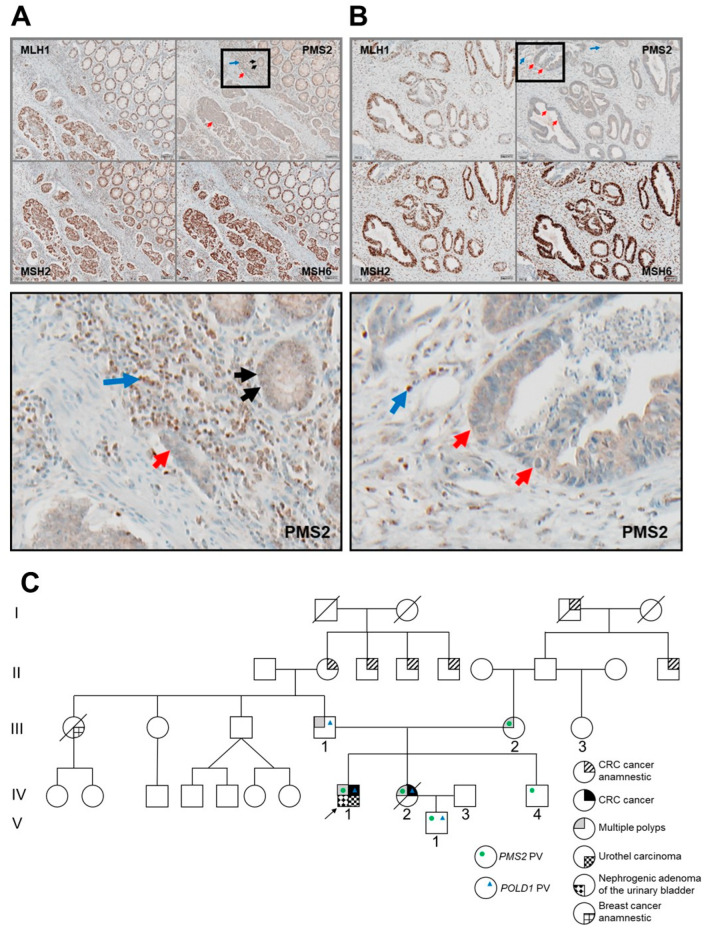
Immunohistochemical staining of mismatch-repair (MMR) proteins (PMS2, MLH1, MSH2 and MSH6) in the tumor of the index patient (**A**) and his sister (**B**). Loss of PMS2 expression is seen in carcinoma epithelia (red arrows) but not in tumor-infiltrating leukocytes (blue arrows; **A**,**B**). PMS2 expression is also absent or strongly reduced in the patient’s non-dysplastic crypts adjacent to carcinoma tissue (black arrows) (**A**). An enlarged view of the relevant areas (black box) is shown below the upper panels (**A**,**B**). In the pedigree of the family (**C**), the identified germline pathogenic variants (PVs), *PMS2*:c.2007-786_2174+493del1447 (green points) and *POLD1*:c.946G>A (blue triangles), are indicated. Cancers are labelled as filled quarters (see figure key). The index patient is depicted by an arrow.

The male index patient had a rectal carcinoma and multiple adenomas at the age of 17 years. He received neoadjuvant chemotherapy, and after abdominoperineal rectal resection, he received adjuvant 5-fluoro-uracil and leucovorin (5-Fu/LV) therapy. Analysis of the carcinoma at the time of diagnosis revealed MSI (Figure S1), but sequencing of *MLH1*, *MSH2* and *APC* was unable to identify a PV in these genes. At age 27 years, a colectomy was performed due to bifocal carcinoma located in the colon ascendens right flexure and in the cecum. The tumor showed isolated PMS2 expression loss in neoplastic cells. Interestingly, PMS2 expression loss was also observed in adjacent non-neoplastic epithelia but not in tumor-infiltrating immune cells (Figure 1A). In agreement with this result, MSI was observed in the rectal carcinoma tissue and in adjacent non-neoplastic tissue (Figure S1). At the age of 34 years, he had a urothelial carcinoma at the distal right ureter, which was partially resected with a uretro-cysto-neostomy. Treatment included three cycles of cisplatin and gemcitabine. Two years later, a nephrogenic adenoma of the urinary bladder was diagnosed and treated with photodynamic diagnosis-assisted transurethral resection and instillation of mitomycin C.

Between the diagnoses of the two CRCs of her brother, the patient’s sister also had synchronous cecal and colon ascendens carcinoma at the age of 19 years. Her cecal carcinoma was immunohistochemically stained and also showed isolated PMS2 expression loss in the neoplastic cells but not in tumor-infiltrating immune cells. Non-dysplastic crypts were not present in this sample (Figure 1B). In addition, she had at least seven polyps, two flat ones close to the ileocecal valve and five small polyp buds in the sigmoid colon and rectum. The sister had a hemicolectomy but died from tumor progression at the age of 21 years. At present, the sibling’s parents, aged 60 and 59 years, respectively, and a brother, aged 35 years, have no history of malignancies. The mother of the patient has had several polyps removed so far during three colonoscopies, including one sessile serrated adenoma, two low-grade tubular adenomas, one hyperplastic polyp, and one high-grade tubulovillous adenoma. The father of the patient had three low-grade tubulovillous adenomas, two in the cecum, and one in the colon descendens, detected during his first surveillance colonoscopy at the age of 57 years. The brother reported a negative screening colonoscopy performed at the age of 34 years. The parents report CRC in the paternal grandmother at the age of 50–60 years and in her three brothers as well as in two third-degree relatives in the maternal line (Figure 1C). However, these anamnestic reports are partially denied by these relatives and cannot be verified.

### 3.2. Identification of Germline PMS2 and POLD1 Variants Causing a Digenic Inheritance of AYA-CRC

**Figure 2:**
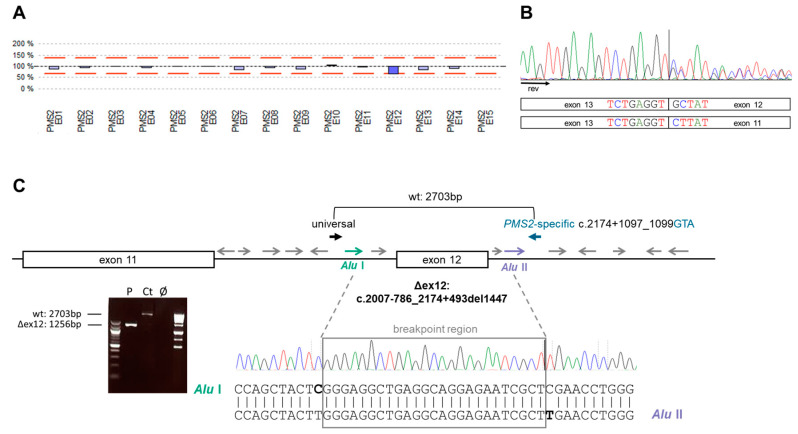
Identification and characterization of the familial *PMS2* exon 12 deletion. Copy number (CN) CN analysis of next-generation sequencing (NGS) data with the SeqNext software shows a loss of one copy of exon 12 of either the *PMS2* gene or the *PMS2CL* pseudogene. Bars indicate the relative CN of each *PMS2* exon (shown on the *x*-axis) in the patient compared to 12 controls. Red lines indicate thresholds for CN variant calling (**A**). Direct *PMS2* gene-specific cDNA sequencing using a reverse primer located in exon 13 (black arrow) shows exon 12 skipping in 50% of the transcripts (**B**). Sequencing of the deletion-spanning gene-specific amplicon reveals an Alu-mediated 1447 bp-deletion (Δex12). These (green and purple) and other (gray) Alu elements in the introns are indicated as arrows in the schematic illustration of *PMS2* exons 11 and 12 and flanking intronic sequences. The amplified region using an unspecific, i.e., not discriminating between *PMS2* and *PMS2CL*, forward primer (black arrow, universal) and a *PMS2*-specific reverse primer (blue arrow) is indicated above the scheme. The shortened 1256 bp PCR amplicon generated from the patient’s DNA and the wild-type (wt) 2703 bp amplicon generated from a control (Ct) DNA (Ø: negative control) are visible in the agarose gel shown on the left below the scheme. Sanger sequencing of the deletion-spanning amplicon of the patient shows a transition from AluI (green) to AluII (purple). The last AluI-specific and the first AluII-specific nucleotides are marked in bold in the sequence shown right below the scheme. The intervening 25 bp sequence in which AluI does not differ from AluII is framed in gray (**C**).

The siblings’ age at CRC diagnosis, the family history with an absence of early onset malignancies in both parents, and the PMS2 expression loss in the neoplastic cells of both siblings’ tumors and in non-neoplastic cells of the index patient strongly suggested that they have CMMRD, although they had no non-neoplastic CMMRD features and PMS2 was expressed in the tumor-infiltrating immune cells of both siblings. To identify the expected CMMRD causing *PMS2* PVs, we performed NGS of the index patient’s blood. A PV was not identified in any of the four MMR genes. However, copy number (CN) analysis of the sequencing data revealed CN loss of *PMS2* (NM_000535.7) exon 12 or the paralogous sequence (with 377 bp of identical sequence) of its pseudogene *PMS2CL* (NR_002217.1) (Figure 2A). MLPA confirmed loss of one of four *PMS2*/*PMS2CL* exon 12 copies but was unable to discern whether this deletion affects the functional *PMS2* gene or the *PMS2CL* pseudogene as the deletion does not extend to the binding site of the paralog-discriminating MLPA probes at position c.2174+1097_2174+1099 in intron 12 (Figure S2). Gene-specific direct cDNA sequencing [21] confirmed a heterozygous exon 12 loss in *PMS2* transcripts (r.2007_2174del; p.Ser669_Ala725delinsArg) (Figure 2B). Subsequent characterization of the deletion breakpoints showed that c.2007-786_2174+493del1447 resulted from non-allelic homologous recombination between two *Alu* elements flanking the *PMS2* exon 12 (Figure 2C). The *PMS2* exon 12 deletion is classified as pathogenic and was also identified by deletion-specific PCR in the patient’s sister’s germline and, hence, explains why the tumors of both siblings were MSI and showed PMS2 expression loss. In view of the low penetrance of *PMS2* PVs [36] and its presence in the germlines of their mother and brother who do not have cancer (Figure 1C), it was deemed unlikely that heterozygosity for this *PMS2* PV was alone responsible for the teenage onset of CRC in both siblings. Nonetheless, a second paternally inherited *PMS2* PV was excluded by transcript analysis, which unequivocally showed bi-allelic expression of *PMS2* with no other (potentially) *PMS2* inactivating alteration than heterozygous exon 12 skipping in the index patient. In addition, a highly sensitive constitutional MSI assay, which detects increased MSI in peripheral blood leukocytes as a pathognomonic feature of CMMRD [22], excluded CMMRD in the index patient.

To explore other or additional causes for the sibling’s phenotype, we expanded the analysis to further genes associated with hereditary CRC and/or polyposis. Sequence and CN analysis of the genes *POLE, POLD1, MSH3, NTHL1, PTEN* (MIM *601728), *STK11* (MIM *602216), *BMPR1A* (MIM *601299) and *SMAD4* (MIM *600993), revealed a single potential PV, NM_002691.4 (*POLD1*):c.946G>A, predicted to cause the amino acid change NP_002682.2 (POLD1):p.(Asp316Asn). This *POLD1* variant alters one of the two exonuclease catalytic residues of POL δ. It is classified as a variant of unknown significance by ClinVar, but it was recently reported as PPAP-causing in a patient with endometrial carcinoma at the age of 54 years and polyposis at the age of 58 years. The latter patient’s father and grandfather (not proven to carry the *POLD1* variant) had CRC (age at diagnosis 45 and 58 years, respectively) and two sisters, one of whom a proven carrier of the *POLD1* variant, had breast cancer (age at diagnosis 53 and 52 years, respectively) [15]. Other missense variants affecting the same amino acid, p.(D316G) and p.(D316H), have also been reported to cause PPAP [15,37]. Following ACMG/AMP guidelines adapted by Mur et al. [18], *POLD1* p.(D316N) is classified as likely pathogenic (LP) based on the following criteria: (i) the variant is located in the exonuclease domain and within the DNA binding cleft (PM1), (ii) it is absent in population database gnomAD [38] (PM2), (iii) it renders a REVEL score of 0.587 [39] that is ≥0.35 (PP3) and (iv) at least one MMRd tumor in the COSMIC database (TCGA-ED-A3KX) [40] and the MMRd tumors of the two siblings—all without any other (suspected) PV in *POLD1*—showed hyper-/ultramutation and the *POLD1*-associated mutational signature SBS20 (see results of tumor sequencing below) (PP4_moderate).

Analysis of our index patient’s family members showed that this *POLD1* variant is paternally inherited and also present in the sister but not in the brother without malignancy (Figure 1C). Together, these findings strongly suggest that the interplay of this *POLD1* variant, likely to impair the proofreading function of POL δ, and the heterozygous *PMS2* PV is responsible for the siblings’ AYA colorectal and other cancers.

### 3.3. Tumor Mutation Characterization Identifies MMRd as an Early Event in Combined MMR and PP Deficiency-Driven Colorectal Tumorigenesis

**Figure 3:**
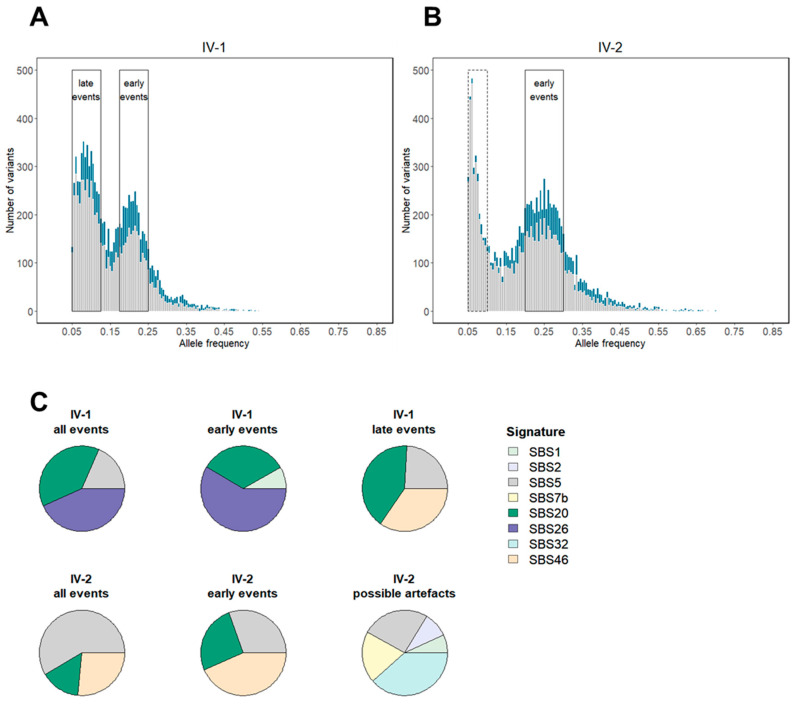
Histogram of somatic single nucleotide variants (SNVs) detected in the patient’s (IV-1; (**A**)) and his sister’s (IV-2; (**B**)) tumor. Short tandem repeat variants are highlighted in dark turquoise. Late and early events with low and high variant allele frequencies (VAFs), respectively, are shown as framed solid. Possible artefacts are framed dashed (**A**,**B**). Pie chart of the signature contribution of all SNVs for the patient’s (IV-1 all events) and his sister’s (IV-2 all events) tumor and signature contribution of separately analyzed late and early SNV events for the patient’s tumor (IV-1 late events; IV-1 early events). For the separate analysis of late and early events, regions suspected to be affected by CN variants and/or loss of heterozygosity (LOH) events were omitted (see Methods and Figure S3A,B) (**C**).

To support that an interaction of a constitutional PP defect and a high propensity to MMRd due to the heterozygous germline *PMS2* PV determines the siblings’ phenotype, we analyzed the TMB and mutational signatures of the patient’s colon and his sister’s cecum carcinoma by whole-exome sequencing. Consistent with PPd, which is characterized by a very high mutation load [41], both tumors were ultra-mutated (TMB: 278 and 319 Mut/Mb for the brother’s and sister’s tumor, respectively). In addition, both tumors have a high proportion of short-tandem-repeat variants, i.e., 27% and 23% for the brother’s and sister’s tumor, respectively, which is typical for (*PMS2*-associated) MSI tumors [41,42] (Figure 3A,B). A high contribution of signature SBS20 in both tumors (38% and 15% in the brother‘s and sister’s tumor, respectively) is in agreement with combined MMR and Pol δ proofreading deficiency (Figure 3C). Importantly, no other somatic PVs in *POLD1* or *POLE* were detected in the siblings’ tumors, supporting that the constitutional *POLD1* p.(D316N) variant is the cause of the combined PPd- and MMRd-specific mutational signatures.

The mutational profile of the index patients’ tumor also showed a high contribution of signature SBS26 (38%), which was recently found to be specific for PMS2 deficiency [43,44]. However, this signature or a different solely MMRd-derived signature, such as SBS6, SBS15, SBS21 or SBS44, is missing in the sister’s tumor. Analysis of gene-specific mutations did not find any somatic second hit point mutations in *PMS2* in either patients’ tumor. Instead, the PMS2 deficiency of both appears to be caused by loss of heterozygosity (LOH)/allelic imbalance (AI) at this locus based on changes in VAF between blood and tumor of known polymorphisms in *PMS2* and adjacent genes, including *SDK1* (MIM *607216), *RBAK-RBAKDN*, *TNRC18*, *AIMP2* (MIM *600859), *USP42* and *ZNF12* (MIM *194536) (Table S1).

The index patients’ tumors clearly showed two mutational bursts with somatic VAFs of 17.5–25% and 5–12.5%, in line with early and late mutational events, respectively (Figure 3A and Figure S3A). Omitting variants on chromosome 15, which exhibits a CN variation event or CN-neutral LOH (Figure S2A), and X and Y chromosomes, the early and the late events include 2891 and 4186 single nucleotide variants (SNVs), respectively. SBS26 contributed to 59% of early mutational events, and SBS20 contributed to 33%. A slightly higher proportion of late mutational events were attributable to SBS20 (41.2%), suggesting combined MMRd and PPd contributed to both early and late mutational events, but no contribution of signature SBS26 was detected, indicating PMS2 deficiency alone drove mutation only in early tumorigenesis (Figure 3C; IV-1).

In the sister’s tumor, two mutational bursts with somatic VAFs of 5–10% and 20–30% can also be seen, again consistent with late and early mutational events, respectively (Figure 3B and Figure S3B). Omitting variants on chromosomes 5, 7, 8, 13, 18 and 19, which exhibit a CN variation event or CN-neutral LOH (Figure S3B), and the X chromosomes, the early events include 3161 and the late events 2158 somatic variants. A significant proportion (26%) of early events can be attributed to SBS20, but the late events were associated with multiple signatures with no known etiology (Figure 3C; IV-2). It is possible, therefore, that the late events are enriched for sequencing or FFPE processing artefacts, which would also fit with the low allele frequency peak at 6% for this burst, which is just above the 5% VAF threshold.

Both tumors, the patient’s colon and his sister’s cecum carcinoma, were further analyzed for somatic PVs in 144 genes related to CRC and polyposis [4,11,45,46,47,48,49,50] (Table S2A). In total, 129 and 164 somatic variants in these genes, of which in each case 11 are considered (likely) pathogenic ((L)P), were detected in the patient’s and his sister’s tumor, respectively. *APC* (MIM *611731), *ACVR2A* (MIM *102581), *KRAS* (MIM *190070) and *ARID1A* (MIM *603024) are frequently mutated in MSI AYA-CRCs [4]. Both tumors have multiple *APC* (L)PVs. In agreement with MMRd being the early driving force in the index patient’s tumor, four of the five identified *APC* variants are frameshift mutations, including the two *APC* (L)PVs falling in the early mutational burst (VAF of >17.5%), whereas four of five *APC* (L)PVs of the sister’s tumor are SNVs. The sister’s tumor had one PV in *ACVR2A*, the most frequently mutated gene in MSI AYA-CRCs. However, no (L)PVs were detected in the other two most frequently mutated genes, *KRAS* and *ARID1A*, in either tumor (Table S2B,C). In the sister’s tumor, *BRAF* c.1799T>A p.(V600E) was found (Table S2C). This mutation has so far not been detected in MSI AYA-CRC and only once in an MSS AYA-CRC [4], and is usually associated with somatic *MLH1* promoter hypermethylation in elderly MSI CRC patients, though it has been infrequently observed in LS CRCs diagnosed <50 years of age [51].

## 4. Conclusions

The two siblings described here are the first reported AYA-CRC cases caused by the concurrence of heterozygous germline PVs in the exonuclease domain of *POLD1* and the MMR gene *PMS2*. *PMS2*-associated CMMRD was initially suspected, which led to the detection of the germline heterozygous *PMS2* exon 12 deletion c.2007-786_2174+493del. Due to high sequence conservation between this region of *PMS2* and its *PMS2CL* pseudogene, it was identified as a loss of one of four copies by NGS and MLPA analyses, requiring cDNA sequencing to resolve its origin. If only poor quality DNA from FFPE tumor tissue was analysed, this variant may have been missed, which could also hold true for other (CN) variants in the notoriously difficult-to-analyse *PMS2* gene. Hence, false negative results should be taken into account when analysing *PMS2* by massively parallel sequencing [52]. In contrast, heterozygous exonuclease mutations in *POLD1* and *POLE* are readily detected by sequencing tumor or germline DNA. However, their classification can be challenging. Using ACMG/AMP guidelines adapted to facilitate the classification of *POLD1* and *POLE* variants [18], the paternally inherited *POLD1* p.(D316N) variant was classified as LP. Taken together, the clinical and genetic findings in the entire family, the exclusion of CMMRD syndrome by absence of MSI in blood leukocytes [22], and the tumor mutational phenotype render overwhelming evidence of a digenic CPS due to the *PMS2* and *POLD1* (L)PVs in the siblings.

There are two previously published cases of childhood/AYA cancer caused by digenic inheritance of a heterozygous *PMS2* PV and a heterozygous *POLE* exonuclease PV. The first describes a patient with a paternally inherited *PMS2* PV c.2174+1G>A, which causes a splicing defect [53], and a maternally inherited *POLE* PV c.890C>G p.(S297C), who presented with synchronous bifocal CRC at the age of 16 years and a non-invasive high-grade urothelial carcinoma at the age of 19 years [54]. Similar to our siblings, PPAP was not suspected in the maternal family prior to identification of the *POLE* PV, although the patient had multiple maternal second-degree relatives with CRC at an age <60 years. The second published case describes a patient born into a known PPAP family carrying the familial *POLE* PV c.830A>G (p.E277G), who presented with a Sonic Hedgehog-activated medulloblastoma at 4.5 years of age [55]. Tumor and subsequent germline analysis revealed that the patient inherited the familial *POLE* PV from his mother and had a de novo germline *PMS2* PV c.2148dupC p.(V717Rfs*19). Taken together, these cases constitute four patients with a digenic inheritance of a *POLE*/*POLD1* PV and an MMR gene PV and an exceptionally young age of cancer onset. Hence, digenic inheritance should be considered and systematically analyzed in all childhood/AYA cancer patients in whom an identified heterozygous germline MMR gene or *POLE*/*POLD1* PV does not alone explain the age of tumor onset and/or phenotype.

The index patient and his nephew, who inherited both PVs in *POLD1* and *PMS2* from the deceased sister (Figure 1C), require clinical surveillance. Currently, surveillance can only be based on clinical findings in the four reported patients with this novel digenic CPS as well as guidelines for LS, PPAP, and CMMRD. AYA-CRC was reported in three of the four, and hence the index patient’s nephew has begun annual colonoscopic surveillance at 12 years of age, which has thus far revealed an unremarkable result. Our index patient and the patient described by Berrino et al. [54] had urothelial carcinoma, a cancer entity that has so far not been described in PPAP [15] but has been in LS, albeit very rarely in *PMS2* PV carriers [36]. Urinary tract cancer surveillance is controversial due to uncertain screening method effectiveness in the context of LS [56]. If offered, it should probably start when the patient is a teenager, given the diagnosis at the age of 19 years in the patient of Berrino et al. [54], and should be performed in a clinical trial of an LS expert center [57]. Childhood medulloblastoma, found in the patient reported by Michaeli et al. [55] at age 4.5 years, is not commonly associated with LS or PPAP but is found in approximately 5% of CMMRD patients [14]. It has also been observed twice in patients with a CMMRD-like phenotype caused by specific, highly penetrant germline *POLE* PVs that appear to confer a stronger “mutator” effect than known PPAP-associated *POLE* PVs [16]. Thus, it is possible that childhood medulloblastoma is specifically associated with the germline *POLE* p.(E277G) variant in the digenic CPS case of Michaeli et al. [55], who also presented with CMMRD-like skin CALMs (as did all of his family members who carried this *POLE* variant) and had an MSS tumor suggesting a strong mutational effect of the *POLE* variant E277G [58]. The risk for childhood brain tumors may therefore be substantially lower in affected members of our case and the patient reported by Berrino et al. [54]. However, brain tumor surveillance may be considered in all (adult) patients with digenic inheritance of an MMR gene PV and *POLD1* or *POLE* PV, as high-grade gliomas have been found in patients with LS [36] and PPAP [15].

The CRCs described in this case were both hypermutated and had MSI. It was recently speculated that cells with *POLD1* PVs acquire a hypermutated phenotype only with concurrent MMRd given that, in contrast to *POLE* PVs, which are found in both MSI and MSS hypermutated tumors, *POLD1* PVs have so far been found only in MSI hypermutated tumors [19]. Of note, the *POLD1* PV p.D316N was observed in two (potentially MMR proficient) tumors with a low TMB (2.7 Mut/Mb) [59]. This may be explained by Pol δ being the main polymerase of the lagging strand on which MMR is more efficient [60]. Nonetheless, a possible mechanism by which constitutional PPd can interact with a heterozygous germline MMR gene defect to cause AYA-CRC is through a high propensity to somatic MMRd from *PMS2* hits due to an increased constitutional mutation rate. As defective PP is associated with an increase in single base exchanges and 1-bp insertions/deletions [58,61], one would expect that SNVs would likely cause somatic *PMS2* inactivation in tumors of these patients. However, in the CRCs of both our index patient and his sister, we could not detect any somatic second hit point mutation in *PMS2* but identified LOH as the mechanism by which the wild-type *PMS2* allele was inactivated. Mutational signature analysis suggests that *PMS2* loss was an early event in both the index patient and sister’s CRCs, with early mutations in both being characterized by SBS20, which is associated with combined MMR- and Pol δ proofreading deficiency [40], with early mutations in the former also being characterized by SBS26, which is associated with PMS2 deficiency alone [43,44]. This is notable since LS colorectal tumorigenesis is generally thought to follow the classic adenoma-carcinoma sequence, whereby MMRd is a late event in adenomas, with established Wnt signaling activation due to somatic *APC*, *CTNNB*1 or *RNF43* PVs, that causes rapid progression to carcinoma [6,62]. However, more recent evidence suggests MMRd is frequently an early, perhaps initiating, event in LS colorectal tumorigenesis. For example, MMRd crypt foci showing loss of expression of the germline-affected MMR gene are found in the otherwise normal colorectal mucosa of LS carriers [63] and can be directly adjacent to MMRd dysfunctional tissue or neoplasia [47,63]. Furthermore, the spectra of mutations in *APC* of LS CRCs were correlated with MMRd mutational signatures in two independent studies [47,64]. Although further studies [65,66] indicate that in patients with *PMS2*-associated LS, MMRd is not an initiating step in CRC pathogenesis, several characteristics of the early-MMRd pathway of LS colorectal tumorigenesis were observed in the CRC of our index patient. MMRd was detected in adjacent normal colorectal mucosa, and in the tumor, mainly somatic frameshift variants in *APC* were found, which likely result from unrepaired insertion/deletion loops due to MMRd. Other mutations frequently found in LS-CRC with early MMRd, such as the hotspot KRAS mutations (G12D and G13D) [47,65], were absent in both tumors. All data taken together, the comprehensive molecular analysis of the patients’ tumors suggest that both developed with MMRd as an early event. However, the loss of PMS2 function was caused by a PPd-independent mechanism consistent with *POLD1* PVs causing hypermutation only with concurrent MMRd [19].

In summary, the case presented here illustrates the importance of considering digenic inheritance in AYA colorectal and other cancer patients, with the concurrence of a heterozygous germline MMR PV and constitutional PPd promoting tumorigenesis early in life. Surveillance for this novel digenic CPS can be derived from the tumor spectrum of related CPS such as LS, PPAP, and CMMRD, but further case descriptions are needed to provide comprehensive recommendations. Our data of the siblings’ CRCs support the idea that MMRd is a prerequisite for a Pol δ defect leading to an ultra-mutated tumor.

## Data Availability

Tumor sequencing data are available upon request.

## Supplementary Materials

The following supporting information can be downloaded at: https://www.mdpi.com/article/10.3390/biom12101350/s1, Figure S1: Microsatellite instability (MSI) analysis of the patient’s neoplastic and non neoplastic cells, both exhibiting PMS2 expression loss; Figure S2: Multiplex ligation dependent probe amplification (MLPA) analysis result shown as relative signal intensities in percent of each MLPA probe in the patient compared to 6 controls demonstrates a 25% (1 of 4 copies) reduction of PMS2 and PMS2CL exon 12 sequences (unspecific: G+P; black solid frame); Figure S3: VAF of somatic variants passing the quality filter (see methods) of the patient’s tumor (IV-1; A) and his sister’s tumor (IV-2; B). Table S1: Variant allele frequency (VAF) in percent of polymorphisms in the PMS2 and flanking genes in blood and tumor tissue of the patients and his sister. Colorcode: low values (red) to high values (blue); Table S2A: Colorectal cancer (CRC)-/Polyposis-associated genes and related pathways with relevance for CRC/Polyposis and DNA-repair pathways; Table S2B: Somatic variants in genes with relevance for CRC/Polyposis and DNA-repair pathways detected in the patient’s tumor; Table S2C: Somatic variants in genes with relevance for CRC/Polyposis and DNA-repair pathways detected in the patient sister’s tumor; Table S3: Supplementary methods.

## References

[1] Siegel R.L., Miller K.D., Fuchs H.E., Jemal A. (2021). Cancer Statistics, 2021. CA Cancer J. Clin..

[2] American Cancer Society Colorectal Cancer Facts & Figures 2017–2019. https://www.cancer.org/research/cancer-facts-statistics/colorectal-cancer-facts-figures.html.

[3] Sultan I., Rodriguez-Galindo C., El-Taani H., Pastore G., Casanova M., Gallino G., Ferrari A. (2010). Distinct features of colorectal cancer in children and adolescents: A population-based study of 159 cases. Cancer.

[4] De Voer R.M., Diets I.J., van der Post R.S., Weren R.D.A., Kamping E.J., de Bitter T.J.J., Elze L., Verhoeven R.H.A., Vink-Börger E., Eijkelenboom A. (2021). Clinical, Pathology, Genetic, and Molecular Features of Colorectal Tumors in Adolescents and Adults 25 Years or Younger. Clin. Gastroenterol. Hepatol..

[5] Burt R. (2007). Inheritance of Colorectal Cancer. Drug Discov. Today Dis. Mech..

[6] Fearon E.R. (2011). Molecular genetics of colorectal cancer. Annu. Rev. Pathol..

[7] Mork M.E., You Y.N., Ying J., Bannon S.A., Lynch P.M., Rodriguez-Bigas M.A., Vilar E. (2015). High Prevalence of Hereditary Cancer Syndromes in Adolescents and Young Adults with Colorectal Cancer. J. Clin. Oncol..

[8] Khan S.A., Morris M., Idrees K., Gimbel M.I., Rosenberg S., Zeng Z., Li F., Gan G., Shia J., LaQuaglia M.P. (2016). Colorectal cancer in the very young: A comparative study of tumor markers, pathology and survival in early onset and adult onset patients. J. Pediatr. Surg..

[9] Fernandez-Rozadilla C., Alvarez-Barona M., Schamschula E., Bodo S., Lopez-Novo A., Dacal A., Calviño-Costas C., Lancho A., Amigo J., Bello X. (2019). Early Colorectal Cancers Provide New Evidence for a Lynch Syndrome-to-CMMRD Phenotypic Continuum. Cancers.

[10] Talseth-Palmer B.A. (2017). The genetic basis of colonic adenomatous polyposis syndromes. Hered. Cancer Clin. Pract..

[11] Jongmans M.C.J., Zhang J., Hoogerbrugge N., Ligtenberg M.J.L., De Voer R.M. (2021). Genetic Cancer Susceptibility in Adolescents and Adults 25 Years or Younger with Colorectal Cancer. Gastroenterology.

[12] Salo-Mullen E.E., Maio A., Mukherjee S., Bandlamudi C., Shia J., Kemel Y., Cadoo K.A., Liu Y., Carlo M., Ranganathan M. (2021). Prevalence and Characterization of Biallelic and Monoallelic NTHL1 and MSH3 Variant Carriers from a Pan-Cancer Patient Population. JCO Precis. Oncol..

[13] Wimmer K., Kratz C.P., Vasen H.F., Caron O., Colas C., Entz-Werle N., Gerdes A.M., Goldberg Y., Ilencikova D., Muleris M. (2014). Diagnostic criteria for constitutional mismatch repair deficiency syndrome: Suggestions of the European consortium ‘care for CMMRD’ (C4CMMRD). J. Med. Genet..

[14] Wimmer K., Rosenbaum T., Messiaen L. (2017). Connections between constitutional mismatch repair deficiency syndrome and neurofibromatosis type 1. Clin. Genet..

[15] Palles C., Martin L., Domingo E., Chegwidden L., McGuire J., Cuthill V., Heitzer E., Kerr R., Kerr D., Kearsey S. (2021). The clinical features of polymerase proof-reading associated polyposis (PPAP) and recommendations for patient management. Fam. Cancer.

[16] Sehested A., Meade J., Scheie D., Østrup O., Bertelsen B., Misiakou M.A., Sarosiek T., Kessler E., Melchior L.C., Munch-Petersen H.F. (2022). Constitutional POLE variants causing a phenotype reminiscent of constitutional mismatch repair deficiency. Hum. Mutat..

[17] Wimmer K., Beilken A., Nustede R., Ripperger T., Lamottke B., Ure B., Steinmann D., Reineke-Plaass T., Lehmann U., Zschocke J. (2017). A novel germline POLE mutation causes an early onset cancer prone syndrome mimicking constitutional mismatch repair deficiency. Fam. Cancer.

[18] Mur P., García-Mulero S., del Valle J., Magraner-Pardo L., Vidal A., Pineda M., Cinnirella G., Martín-Ramos E., Pons T., López-Doriga A. (2020). Role of POLE and POLD1 in familial cancer. Genet. Med..

[19] Park V.S., Pursell Z.F. (2019). POLE proofreading defects: Contributions to mutagenesis and cancer. DNA Repair.

[20] Heydt C., Fassunke J., Künstlinger H., Ihle M.A., König K., Heukamp L.C., Schildhaus H.U., Odenthal M., Büttner R., Merkelbach-Bruse S. (2014). Comparison of pre-analytical FFPE sample preparation methods and their impact on massively parallel sequencing in routine diagnostics. PLoS ONE.

[21] Etzler J., Peyrl A., Zatkova A., Schildhaus H.U., Ficek A., Merkelbach-Bruse S., Kratz C.P., Attarbaschi A., Hainfellner J.A., Yao S. (2008). RNA-based mutation analysis identifies an unusual MSH6 splicing defect and circumvents PMS2 pseudogene interference. Hum. Mutat..

[22] Gallon R., Mühlegger B., Wenzel S.S., Sheth H., Hayes C., Aretz S., Dahan K., Foulkes W., Kratz C.P., Ripperger T. (2019). A sensitive and scalable microsatellite instability assay to diagnose constitutional mismatch repair deficiency by sequencing of peripheral blood leukocytes. Hum. Mutat..

[23] Hiatt J.B., Pritchard C.C., Salipante S.J., O’Roak B.J., Shendure J. (2013). Single molecule molecular inversion probes for targeted, high-accuracy detection of low-frequency variation. Genome Res..

[24] Li H., Durbin R. (2009). Fast and accurate short read alignment with Burrows-Wheeler transform. Bioinformatics.

[25] Wernstedt A., Valtorta E., Armelao F., Togni R., Girlando S., Baudis M., Heinimann K., Messiaen L., Staehli N., Zschocke J. (2012). Improved multiplex ligation-dependent probe amplification analysis identifies a deleterious PMS2 allele generated by recombination with crossover between PMS2 and PMS2CL. Genes Chromosomes Cancer.

[26] Richards S., Aziz N., Bale S., Bick D., Das S., Gastier-Foster J., Grody W.W., Hegde M., Lyon E., Spector E. (2015). Standards and guidelines for the interpretation of sequence variants: A joint consensus recommendation of the American College of Medical Genetics and Genomics and the Association for Molecular Pathology. Genet. Med..

[27] The InSiGHT Variant Interpretation Committee Mismatch Repair Gene Variant Classification Criteria 2018. https://www.insight-group.org/content/uploads/2018/08/2018-06_InSiGHT_VIC_v2.4.pdf.

[28] Li H., Handsaker B., Wysoker A., Fennell T., Ruan J., Homer N., Marth G., Abecasis G., Durbin R. (2009). The Sequence Alignment/Map format and SAMtools. Bioinformatics.

[29] Köster J., Rahmann S. (2012). Snakemake—A scalable bioinformatics workflow engine. Bioinformatics.

[30] Ewels P., Magnusson M., Lundin S., Käller M. (2016). MultiQC: Summarize analysis results for multiple tools and samples in a single report. Bioinformatics.

[31] McLaren W., Gil L., Hunt S.E., Riat H.S., Ritchie G.R.S., Thormann A., Flicek P., Cunningham F. (2016). The Ensembl Variant Effect Predictor. Genome Biol..

[32] Pedersen B.S., Quinlan A.R. (2017). Mosdepth: Quick coverage calculation for genomes and exomes. Bioinformatics.

[33] Benjamin D., Sato T., Cibulskis K., Getz G., Stewart C., Lichtenstein L. (2019). Calling Somatic SNVs and Indels with Mutect2. bioRxiv.

[34] Andrews S. (2020). S-andrews/FastQC [Java]. https://github.com/s-andrews/FastQC.

[35] Kandoth C. (2020). Mskcc/vcf2maf [Perl]. https://github.com/mskcc/vcf2maf.

[36] Dominguez-Valentin M., Sampson J.R., Seppälä T.T., ten Broeke S.W., Plazzer J.-P., Nakken S., Engel C., Aretz S., Jenkins M.A., Sunde L. (2020). Cancer risks by gene, age, and gender in 6350 carriers ofpathogenic mismatch repair variants: Findings from the Prospective Lynch SyndromeDatabase. Genet. Med..

[37] Bellido F., Pineda M., Aiza G., Valdés-Mas R., Navarro M., Puente D.A., Pons T., González S., Iglesias S., Darder E. (2016). POLE and POLD1 mutations in 529 kindred with familial colorectal cancer and/or polyposis: Review of reported cases and recommendations for genetic testing and surveillance. Genet. Med..

[38] Karczewski K.J., Francioli L.C., Tiao G., Cummings B.B., Alföldi J., Wang Q., Collins R.L., Laricchia K.M., Ganna A., Birnbaum D.P. (2020). The mutational constraint spectrum quantified from variation in 141,456 humans. Nature.

[39] Ioannidis N.M., Rothstein J.H., Pejaver V., Middha S., McDonnell S.K., Baheti S., Musolf A., Li Q., Holzinger E., Karyadi D. (2016). REVEL: An Ensemble Method for Predicting the Pathogenicity of Rare Missense Variants. Am. J. Hum. Genet..

[40] Haradhvala N.J., Kim J., Maruvka Y.E., Polak P., Rosebrock D., Livitz D., Hess J.M., Leshchiner I., Kamburov A., Mouw K.W. (2018). Distinct mutational signatures characterize concurrent loss of polymerase proofreading and mismatch repair. Nat. Commun..

[41] Cancer Genome Atlas Network (2012). Comprehensive molecular characterization of human colon and rectal cancer. Nature.

[42] Bajwa-Ten Broeke S.W., Ballhausen A., Ahadova A., Suerink M., Bohaumilitzky L., Seidler F., Morreau H., van Wezel T., Krzykalla J., Benner A. (2021). The coding microsatellite mutation profile of PMS2-deficient colorectal cancer. Exp. Mol. Pathol..

[43] Zou X., Koh G.C.C., Nanda A.S., Degasperi A., Urgo K., Roumeliotis T.I., Agu C.A., Badja C., Momen S., Young J. (2021). A systematic CRISPR screen defines mutational mechanisms underpinning signatures caused by replication errors and endogenous DNA damage. Nat. Cancer.

[44] Koh G., Degasperi A., Zou X., Momen S., Nik-Zainal S. (2021). Mutational signatures: Emerging concepts, caveats and clinical applications. Nat. Rev. Cancer.

[45] Krokan H.E., Bjørås M. (2013). Base excision repair. Cold Spring Harb. Perspect Biol..

[46] Mouradov D., Sloggett C., Jorissen R.N., Love C.G., Li S., Burgess A.W., Arango D., Strausberg R.L., Buchanan D., Wormald S. (2014). Colorectal cancer cell lines are representative models of the main molecular subtypes of primary cancer. Cancer Res..

[47] Ahadova A., Gallon R., Gebert J., Ballhausen A., Endris V., Kirchner M., Stenzinger A., Burn J., von Knebel Doeberitz M., Bläker H. (2018). Three molecular pathways model colorectal carcinogenesis in Lynch syndrome. Int. J. Cancer.

[48] Stanich P.P., Pearlman R., Hinton A., Gutierrez S., LaDuca H., Hampel H., Jasperson K. (2019). Prevalence of Germline Mutations in Polyposis and Colorectal Cancer-Associated Genes in Patients with Multiple Colorectal Polyps. Clin. Gastroenterol. Hepatol..

[49] Jelsig A.M., Byrjalsen A., Busk Madsen M., Kuhlmann T.P., van Overeem Hansen T., Wadt K.A.W., Karstensen J.G. (2021). Novel Genetic Causes of Gastrointestinal Polyposis Syndromes. Appl. Clin. Genet..

[50] Lee C.S., Song I.H., Lee A., Kang J., Lee Y.S., Lee I.K., Song Y.S., Lee S.H. (2021). Enhancing the landscape of colorectal cancer using targeted deep sequencing. Sci. Rep..

[51] Bläker H., Haupt S., Morak M., Holinski-Feder E., Arnold A., Horst D., Sieber-Frank J., Seidler F., von Winterfeld M., Alwers E. (2020). Age-dependent performance of BRAF mutation testing in Lynch syndrome diagnostics. Int. J. Cancer.

[52] Mandelker D., Schmidt R.J., Ankala A., McDonald Gibson K., Bowser M., Sharma H., Duffy E., Hegde M., Santani A., Lebo M. (2016). Navigating highly homologous genes in a molecular diagnostic setting: A resource for clinical next-generation sequencing. Genet. Med..

[53] van der Klift H.M., Jansen A.M., van der Steenstraten N., Bik E.C., Tops C.M., Devilee P., Wijnen J.T. (2015). Splicing analysis for exonic and intronic mismatch repair gene variants associated with Lynch syndrome confirms high concordance between minigene assays and patient RNA analyses. Mol. Genet. Genom. Med..

[54] Berrino E., Filippi R., Visintin C., Peirone S., Fenocchio E., Farinea G., Veglio F., Aglietta M., Sapino A., Cereda M. (2022). Collision of germline POLE and PMS2 variants in a young patient treated with immune checkpoint inhibitors. NPJ Precis. Oncol..

[55] Michaeli O., Ladany H., Erez A., Ben Shachar S., Izraeli S., Lidzbarsky G., Basel-Salmon L., Biskup S., Maruvka Y.E., Toledano H. (2022). Di-genic inheritance of germline POLE and PMS2 pathogenic variants causes a unique condition associated with pediatric cancer predisposition. Clin. Genet..

[56] Lonati C., Necchi A., Gómez Rivas J., Afferi L., Laukhtina E., Martini A., Ventimiglia E., Colombo R., Gandaglia G., Salonia A. (2022). Upper Tract Urothelial Carcinoma in the Lynch Syndrome Tumour Spectrum: A Comprehensive Overview from the European Association of Urology-Young Academic Urologists and the Global Society of Rare Genitourinary Tumors. Eur. Urol. Oncol..

[57] Bernstein I.T., Myrhøj T. (2013). Surveillance for urinary tract cancer in Lynch syndrome. Fam. Cancer.

[58] Chung J., Maruvka Y.E., Sudhaman S., Kelly J., Haradhvala N.J., Bianchi V., Edwards M., Forster V.J., Nunes N.M., Galati M.A. (2021). DNA Polymerase and Mismatch Repair Exert Distinct Microsatellite Instability Signatures in Normal and Malignant Human Cells. Cancer Discov..

[59] Campbell B.B., Light N., Fabrizio D., Zatzman M., Fuligni F., de Borja R., Davidson S., Edwards M., Elvin J.A., Hodel K.P. (2017). Comprehensive Analysis of Hypermutation in Human Cancer. Cell.

[60] Andrianova M.A., Bazykin G.A., Nikolaev S.I., Seplyarskiy V.B. (2017). Human mismatch repair system balances mutation rates between strands by removing more mismatches from the lagging strand. Genome Res..

[61] Hodel K.P., de Borja R., Henninger E.E., Campbell B.B., Ungerleider N., Light N., Wu T., LeCompte K.G., Goksenin A.Y., Bunnell B.A. (2018). Explosive mutation accumulation triggered by heterozygous human Pol ε proofreading-deficiency is driven by suppression of mismatch repair. Elife.

[62] Lynch H.T., Snyder C.L., Shaw T.G., Heinen C.D., Hitchins M.P. (2015). Milestones of Lynch syndrome: 1895–2015. Nat. Rev. Cancer.

[63] Kloor M., Huth C., Voigt A.Y., Benner A., Schirmacher P., von Knebel Doeberitz M., Bläker H. (2012). Prevalence of mismatch repair-deficient crypt foci in Lynch syndrome: A pathological study. Lancet Oncol..

[64] Sekine S., Mori T., Ogawa R., Tanaka M., Yoshida H., Taniguchi H., Nakajima T., Sugano K., Yoshida T., Kato M. (2017). Mismatch repair deficiency commonly precedes adenoma formation in Lynch Syndrome-Associated colorectal tumorigenesis. Mod. Pathol..

[65] Helderman N.C., Bajwa-Ten Broeke S.W., Morreau H., Suerink M., Terlouw D., van der Werf T.L.A.S., van Wezel T., Nielsen M. (2021). The diverse molecular profiles of lynch syndrome-associated colorectal cancers are (highly) dependent on underlying germline mismatch repair mutations. Crit. Rev. Oncol. Hematol..

[66] Ten Broeke S.W., van Bavel T.C., Jansen A.M.L., Gómez-García E., Hes F.J., van Hest L.P., Letteboer T.G.W., Olderode-Berends M.J.W., Ruano D., Spruijt L. (2018). Molecular Background of Colorectal Tumors from Patients with Lynch Syndrome Associated with Germline Variants in PMS2. Gastroenterology.

