# Supplementary material for: Teenage-Onset Colorectal Cancers in a Digenic Cancer Predisposition Syndrome Provide Clues for the Interaction between Mismatch Repair and Polymerase δ Proofreading Deficiency in Tumorigenesis
Source: Biomolecules. 2022 Sep 22;12(10):1350. doi: 10.3390/biom12101350 (PMC9599501; doi:10.3390/biom12101350)
Supplement: Supplementary file 1 [file biomolecules-12-01350-s001.zip › Figures and Tables/Figure S1.pdf]

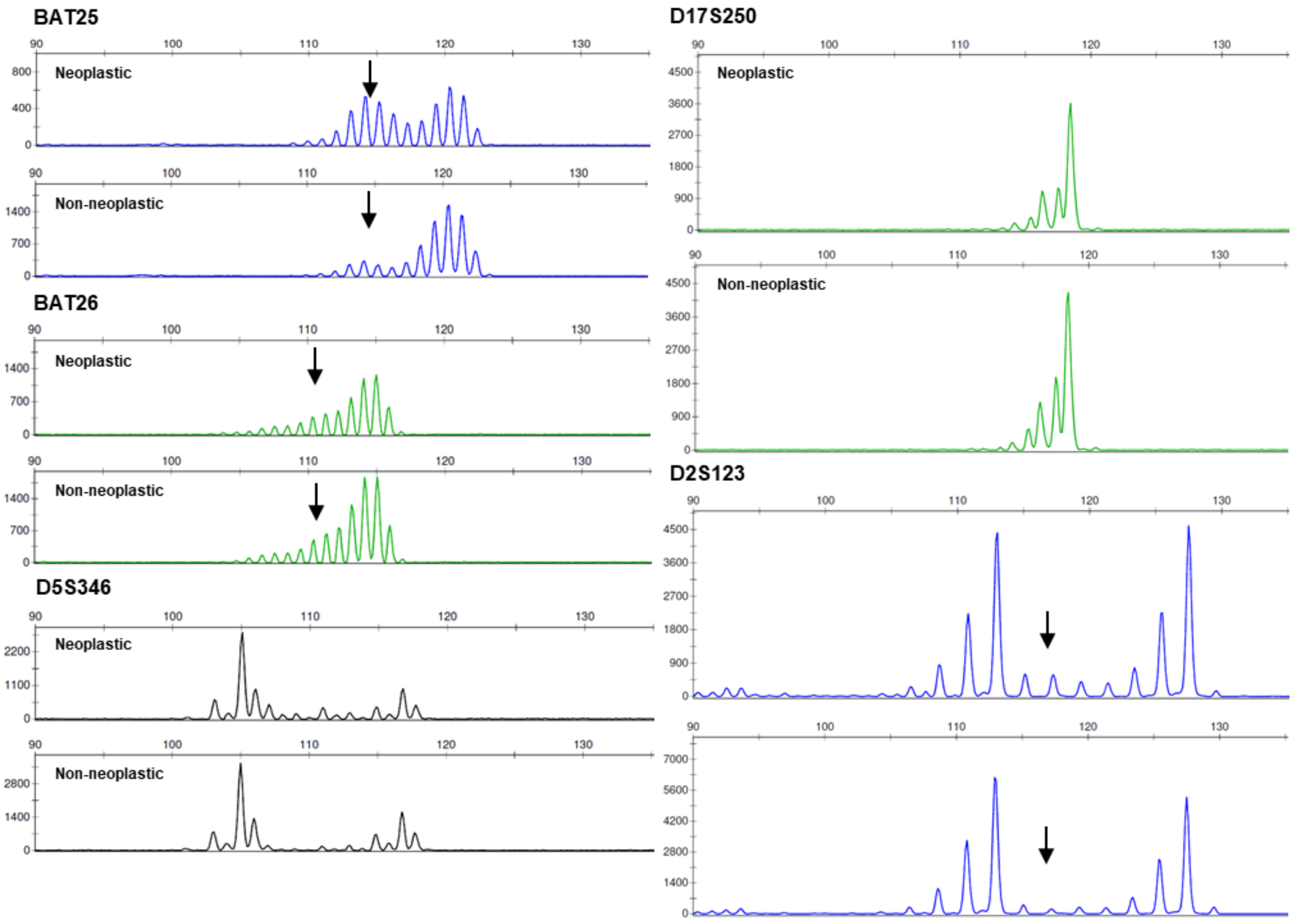

Supplementary Figure S1. Microsatellite instability (MSI) analysis of the patient´s neoplastic and non-neoplastic cells, both exhibiting PMS2 expression loss. Analysis of 5 MSI markers (BAT25, BAT26, D5S346, D2S123 and D17S250) indicates MSI in three markers (BAT25,BAT265 and D2S123) in both the neoplastic and non-neoplastic cells of the patient's tumor. Additional alleles are highlighted by black arrows.
