# Supplementary material for: Teenage-Onset Colorectal Cancers in a Digenic Cancer Predisposition Syndrome Provide Clues for the Interaction between Mismatch Repair and Polymerase δ Proofreading Deficiency in Tumorigenesis
Source: Biomolecules. 2022 Sep 22;12(10):1350. doi: 10.3390/biom12101350 (PMC9599501; doi:10.3390/biom12101350)
Supplement: Supplementary file 1 [file biomolecules-12-01350-s001.zip › Figures and Tables/Figure S2.pdf]

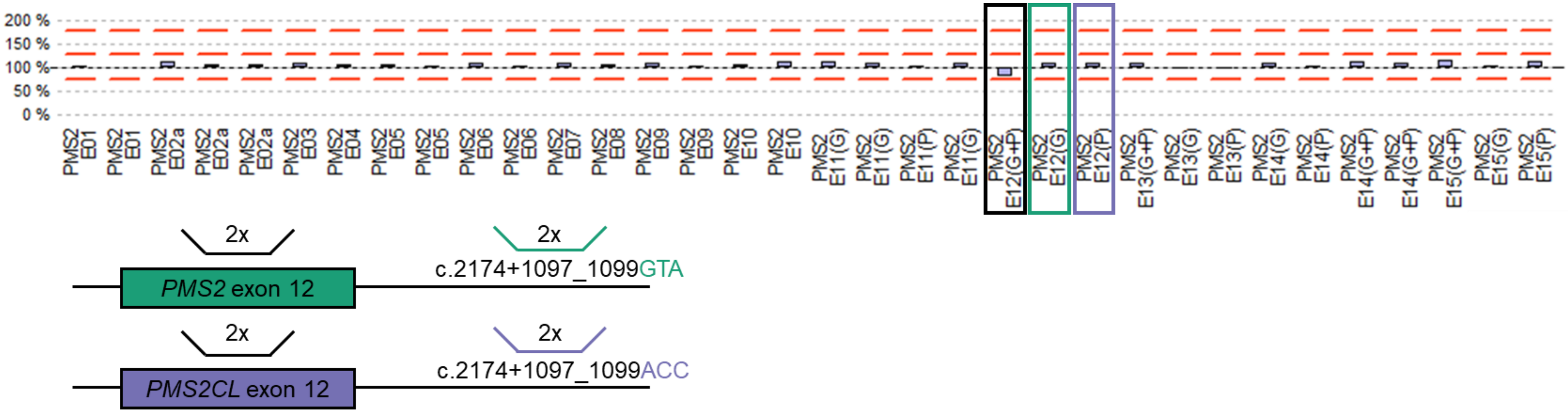

Supplementary Figure S2. Multiplex-ligation-dependent probe amplification (MLPA) analysis result shown as relative signal intensities in percent of each MLPA probe in the patient compared to 6 controls demonstrates a 25% (1 of 4 copies) reduction of *PMS2* and *PMS2CL* exon 12 sequences (unspecific: G +P; black solid frame). Gene (G) and pseudogene (P) specific probes and their location in relation to *PMS2* exon 12 are shown in green and purple, respectively (schematic illustration, bottom). Probe labels are indicated below the plot. Red lines indicate relative signal intensity thresholds for calling variants.
