# Supplementary material for: Teenage-Onset Colorectal Cancers in a Digenic Cancer Predisposition Syndrome Provide Clues for the Interaction between Mismatch Repair and Polymerase δ Proofreading Deficiency in Tumorigenesis
Source: Biomolecules. 2022 Sep 22;12(10):1350. doi: 10.3390/biom12101350 (PMC9599501; doi:10.3390/biom12101350)
Supplement: Supplementary file 1 [file biomolecules-12-01350-s001.zip › Figures and Tables/Figure S3.pdf]

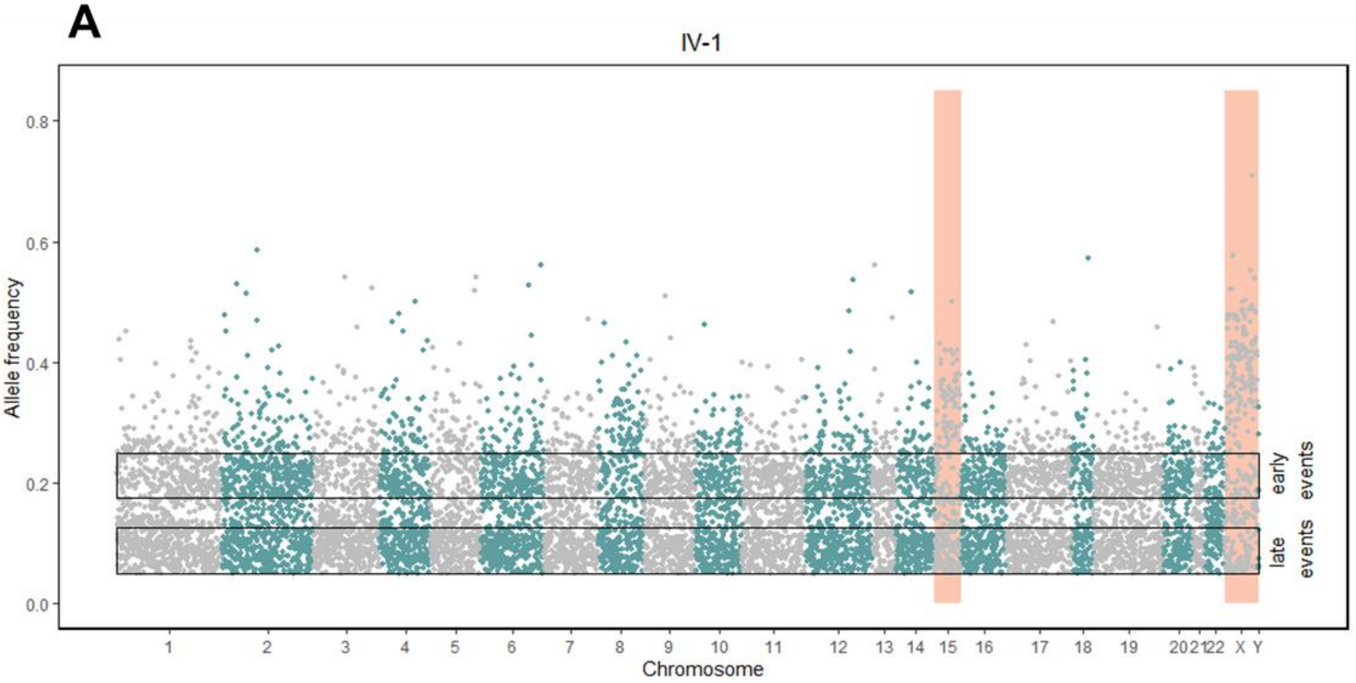

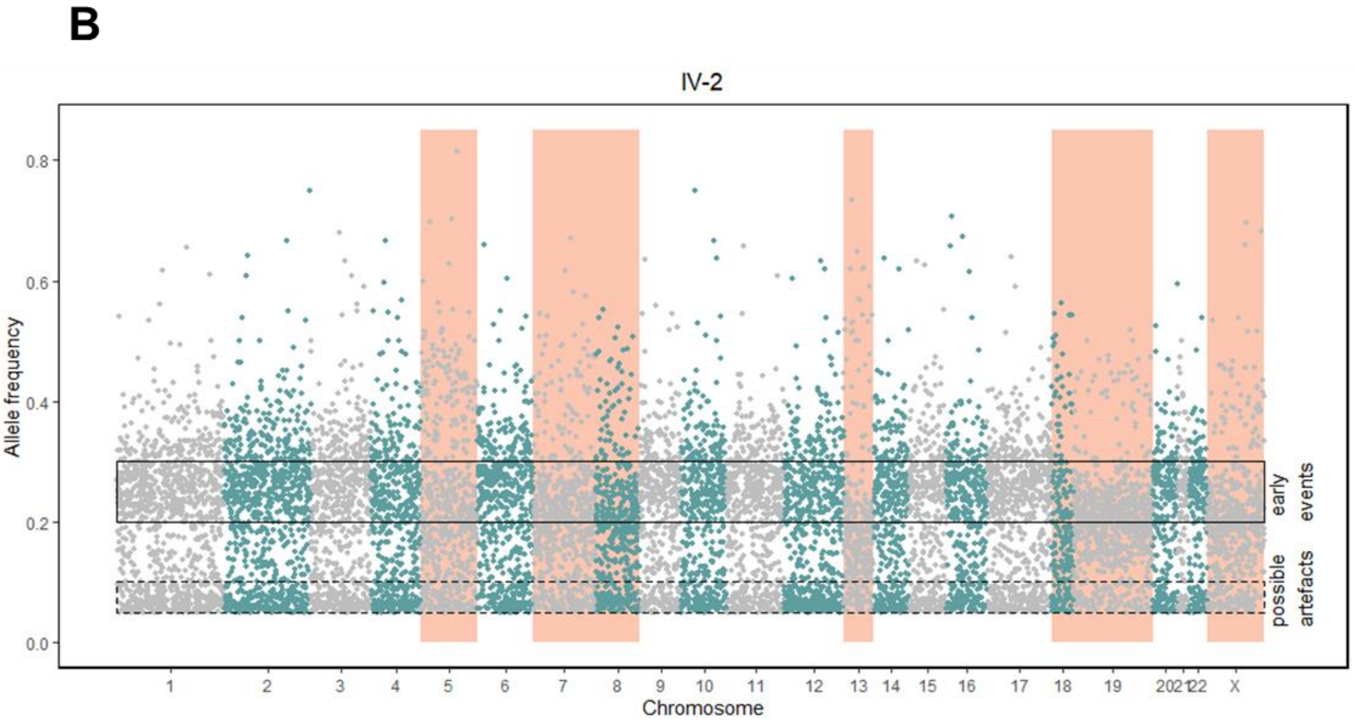

Supplementary Figure S3. VAF of somatic variants passing the quality filter (see methods) of the patient´s tumor (IV-1; A) and his sister´s tumor (IV-2; B). Regions showing CN variation and/or LOH events are marked in salmon color. Variants with VAFs assigned to early and late events in the patient’s tumor are shown in black boxes. Variants with VAF considered possible artefacts are shown in dashed boxes (IV-1, A; IV-2, B). For mutational signature analysis variants located in regions with CN variation and/or LOH (highlighted in salmon) were omitted.
